# Diseases of the Skin

**Published:** 1877-10

**Authors:** 


*An Elementary Treatise on Diseases of the Skin, for the use of Students and Practitioners.* By HENRY G. PIFFARD, A. M., M. D., Prof. of Dermatology, University of New York. With Illustrations. London and New York: Macmillan & Co., 1876. Buffalo: Herger & Ulbrich.

Forty-nine wood cuts and five photo-micrographs embellish and enrich this excellent volume. The photo-micrographs are especially to be commended as life-like and accurate, while the wood cuts are executed in a style that is seldom found in works of this kind, indeed, the typography is unexcelled. As to matter, it is truly modern, an epitome of the latest thought upon the pathology and treatment of skin diseases. The first six chapters are devoted to the consideration of the anatomy, physiology and pathology of the skin; and the symptomatology, diagnosis, and classification of skin diseases. The remainder of the work consists of the history, pathology, diagnosis and treatment of the various diseases as they are approached in the classification. The author makes five classes: 1. Diathetic,—consisting of the syphilides, scrofulides, rheumides, leprosy and ichthyosis. 2. General non-diathetic affections,—eruptive fevers, erysipelas, etc. 3. Reflex affections,—acne, rosacea, etc. 4. Local affection,—parasitic and non-parasitic 5. Affections of uncertain nature, being those which do not arrange themselves under the other classes. In this mode of division he differs from Dr. Bulkley, who makes nine classes: Parasitic, glandular, neurotic, hyperæmic, exudative, hæmorrhagic, hypertrophic, atrophic and new formations; and we must give the latter the credit of the most perfect classification, the diseases falling more naturally under their appropriate head.

The most important and undisputed are placed in large, while the questionable and unimportant, together with all reports of cases, are in small type. Much pains to collect the opinion of others is shown while his own are clearly stated. The language is pure, strong and fluent, and will be found pleasant; altogether we would recommend it to be worthy careful consideration and useful to every physician. C.

